# Intermittent Hypothermia in Hodgkin’s Lymphoma: A Potential Indicator for Urgent Chemotherapy Initiation

**DOI:** 10.1155/carm/5915477

**Published:** 2026-05-26

**Authors:** Phillip Papayanis, Dina Nashed, Paul J. Pecorin, Seo-Hyun Kim, Irene Dehghan-Paz

**Affiliations:** ^1^ Department of Internal Medicine, Rush University Medical Center, Chicago, Illinois, USA, rush.edu

**Keywords:** Hodgkin’s lymphoma, hypothermia, temperature instability

## Abstract

Hypothermia is defined as a body temperature of less than 35°C and has a broad differential diagnosis, including metabolic and infectious causes, as well as malignancy. In this case, we present a patient with Hodgkin’s lymphoma (HL) who developed hypothermia leading to clinical instability requiring admission to an intensive care unit. After ruling out all other causes of hypothermia, we reached the conclusion that HL could be a culprit. This prompted us for urgent inpatient chemotherapy induction, eventually leading to resolution of her temperature instability, which confirmed our hypothesis. We believe our case adds to the small body of literature documenting hypothermia in patients with HL and suggests that HL should be considered on the differential for a patient presenting with hypothermia. Additionally, our case suggests that the presence of hypothermia, causing clinical instability in a patient with HL, may indicate the need for more urgent treatment of HL.

## 1. Introduction

Hypothermia, defined as a core body temperature below 35°C, has a broad differential diagnosis including infectious, metabolic, neurologic, and iatrogenic causes. Management typically involves active rewarming such as warm blankets, warmed intravenous fluids, or body cavity lavage alongside treatment of the underlying cause [1]. Hypothermia is considered as a poor prognostic indicator in multiple pathologies including sepsis where multiple prospective and retrospective trials show that patients with hypothermia have increased mortality compared to those who are normothermic or hyperthermic [2, 3].

In malignancy, hypothermia is rare and poorly understood. There are isolated case reports of fatal hypothermia in patients with glioblastoma multiforme due to hypothalamic involvement, but otherwise, hypothermia in solid tumors is rarely described [4]. In Hodgkin’s lymphoma (HL), however, hypothermia is a rare but documented occurrence, with one source noting around 18 reported cases. Despite multiple case reports, hypothermia remains a poorly understood complication without clear guidelines on management [5]. One explanation could be from medications such as antipyretics, but several of the reported cases had hypothermia occurrences prior to treatment with any of these medications such as acetaminophen or steroids [4, 5]. Other previously proposed mechanisms included direct hypothalamic involvement, cytokine‐mediated hypothalamic hypersensitivity, paraneoplastic phenomena, and autonomic dysfunction [4, 5]. The mechanisms by which these processes lead to hypothermia are complex. The hypothalamus, specifically the preoptic area, serves as the body’s central temperature regulator. Variations in the external temperature or the body temperature active central thermoreceptors that attempt to regulate temperature. In the setting of malignancy, disruption of normal thermoregulation can result in either hypothermia. Malignancy can lead to aberrant production of Interleukin 1 (IL‐1), Interleukin 6 (IL‐6), and tumor necrosis factor‐a (TNF‐a). Although typically regarded as endogenous pyrogens, they can function as cryogens in the setting of inflammation [6]. Additionally, autonomic dysfunction, which is implicated in some paraneoplastic syndromes, can impair appropriate regulation of core body temperature. There is debate regarding whether this dysautonomia is caused by malignancy itself, such as lymphoma, or chemotherapy. But studies suggest that there is a strong associate with the underlying disease process [7, 8].

Here, we present a case of a patient with newly diagnosed HL who developed intermittent hypothermia prior to chemotherapy, which resolved after initiation of treatment. This case highlights the workup of hypothermia in the hospital setting as well as the importance of including HL on the differential of unexplained hypothermia. Additionally, recognizing this rare manifestation may prompt early diagnostic evaluation and serve as an indication for inpatient chemotherapy.

## 2. Case Overview

A 73‐year‐old woman with no significant past medical history presented to the emergency department with one month of progressive fatigue, poor appetite, a 10‐pound weight loss, and outpatient lab work with a hemoglobin of 6.9 g/dL. On presentation, she was hypotensive (90/50 mmHg, MAP 63) with her exam notable for a diffusely tender abdomen. Laboratory studies were notable for sodium 127 mmol/L, alkaline phosphatase 212 U/L, aspartate aminotransferase 84 U/L, alanine aminotransferase 105 U/L, white blood cell count of 3.67 × 10^9^/L, and hemoglobin 8.4 g/dL, which notably was higher than outpatient level without any transfusion.

**FIGURE 1:**
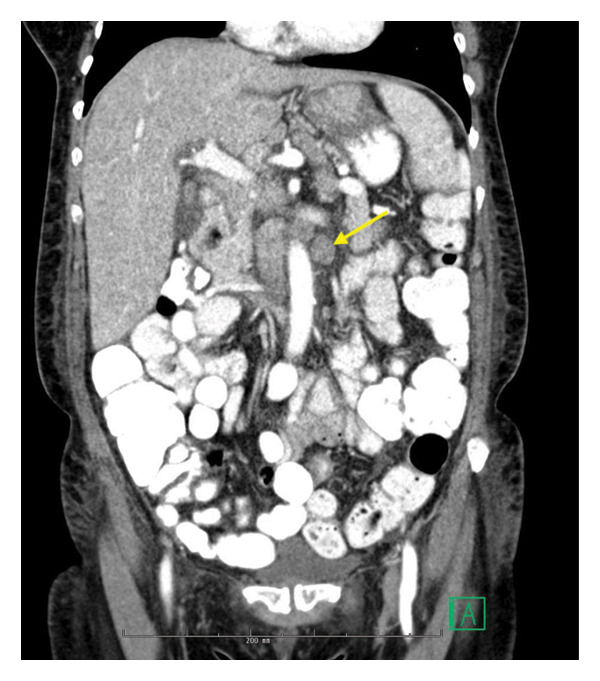
Initial computerized tomography on Day 1 of admission. Arrow noting celiac lymphadenopathy.

CT imaging of the abdomen and pelvis with IV but no oral contrast revealed mild hepatosplenomegaly, diffuse liver and splenic hypodensities, and multiple enlarged retroperitoneal and abdominal lymph nodes (Figure 1), concerning for lymphoma or metastatic malignancy. A CT brain showed mild microvascular ischemic changes without evidence of central nervous system lesions. She was admitted for further management and her hypotension improved with fluid. For further diagnostic workup, an endoscopic biopsy of perigastric lymph nodes was performed on Hospital Day 3.

**FIGURE 2:**
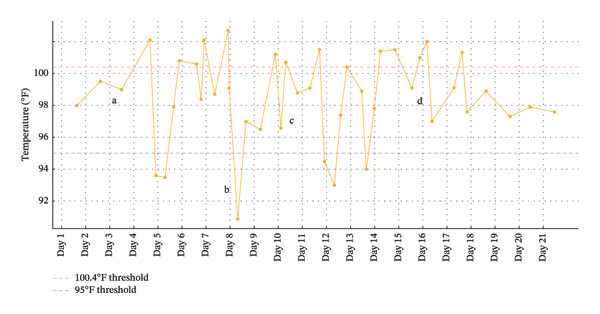
Patient’s fever curve throughout admission. Day 3: (a) initial gastric lymph node biopsy is performed. Day 8: (b) biopsy results come back showing Hodgkin’s lymphoma. Day 10: (c) initial treatment is started with prednisone. Day 16: (d) treatment with doxorubicin, vinblastine, and dacarbazine is started.

On Day 4, the patient developed a fever up to 38.8°C (Figure 2). Infectious workup with blood culture, respiratory pathogen panel, urinalysis, urine culture, and chest X‐ray were unrevealing. Fever was presumed to be malignant as routine infectious workup was negative though rare causes of fevers such as tick‐borne illnesses were not ruled out. The following morning, she became hypothermic to 34.2°C. Infectious workup was again unrevealing, but given continued temperature instability empiric cefepime and metronidazole were initiated. Despite escalation of antibiotics due to ongoing fevers on Days 6 and 7, temperature instability persisted.

On Day 8, biopsy results confirmed classic HL, mixed‐cellularity subtype. Over the next several days, the patient developed worsening hypothermia, with a nadir of 32.7°C, necessitating ICU transfer for active rewarming with a Bair Hugger system and more frequent vital checks. Vancomycin was also added for additional coverage. Additional hypothermia workup was unrevealing including a thyroid‐stimulating hormone of 2.312 µIU/mL and AM cortisol of 17.0 μg/dL. Additionally, a CT scan of her brain did not reveal any lesions that would suggest hypothalamic involvement. After alternative causes of hypothermia were ruled out, prednisone was initiated on Day 10 with plans for outpatient chemotherapy. However, from Days 11–14, the patient continued to experience alternating fevers and hypothermia. Repeat infectious workup remained negative, and shock was deemed unlikely given stable lactate levels and blood pressure.

Given the persistence of symptoms and lack of an alternate explanation, HL‐related autonomic or cytokine‐driven hypothermia was suspected. Due to clinical instability and limited response to prednisone (with multiple episodes of hypothermia and fevers even after 3‐4 days), inpatient chemotherapy was initiated on Day 16 with doxorubicin, vinblastine, and dacarbazine (AVD) plus filgrastim. Nivolumab was planned for outpatient initiation. Following chemotherapy, the patient’s temperature stabilized by Day 18 after the first dose of treatment, with no recurrence of fevers or hypothermia. She was discharged to acute rehabilitation with outpatient hematology follow‐up.

Since discharge, she has completed 6 cycles of nivolumab, doxorubicin, vinblastine, and dacarbazine with good response. Her PET scan 6 months after initiation of treatment showed no significant FDG avid lymphadenopathy. She continues to follow up for surveillance every 3 months.

## 3. Discussion

Hypothermia is a rare and poorly understood complication of HL for which the etiology is unclear, and management recommendations are not well established. In hospitalized patients, hypothermia is associated with poor outcomes and carries a broad differential including infection, endocrinopathies, neurologic causes, and adverse reactions to medications [2]. When hypothermia is identified in a patient with HL, lymphoma‐related causes must also be included in this differential. In our case, other causes of hypothermia were systematically evaluated and excluded. Infectious etiologies were unlikely given negative serial infectious studies, stable hemodynamics, and normal lactate levels. Endocrine causes were excluded by normal thyroid studies and morning cortisol. Medication‐related side effects were unlikely as episodes occurred unrelated to antipyretic administration. Finally, central causes such as hypothalamic lesions were unlikely due to negative brain imaging. Overall, given negative workup, HL‐related etiology was the most likely explanation.

**FIGURE 3:**
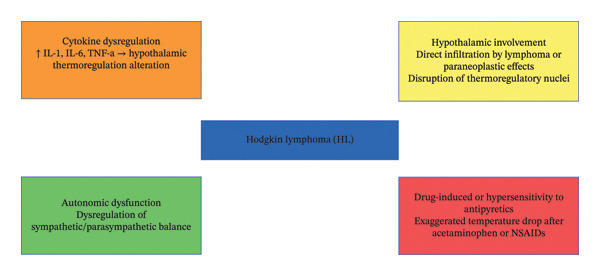
Proposed mechanisms of hypothermia in HL including cytokine dysregulation, hypothalamic involvement of HL, autonomic dysfunction, and antipyretic hypersensitivity.

Our patient exhibited cyclic fevers and profound hypothermia in the absence of infection or other identifiable etiologies. The temperature instability then resolved promptly after the initiation of chemotherapy, strongly suggesting a direct link to her underlying lymphoma. The etiology of hypothermia in HL is not well defined. Autonomic dysfunction, cytokine dysregulation, hypersensitivity to antipyretics, and hypothalamic involvement all have been considered in prior HL cases (Figure 3) [4, 5, 9]. In one case, it is even postulated that a patient’s HL may be producing an insulin‐like substance or insulin autoantibody [7]. Overall, the literature on the topic consists of entirely cases and case series [4, 5, 7]. In this case, our patient had a cyclic and intermittent pattern of hypothermia with onset prior to chemotherapy and resolution with treatment of her HL, making cytokine dysregulation and autonomic dysfunction plausible etiologies. Antipyretic hypersensitivity can also be considered; however, this is less likely, as at least one episode was in the absence of antipyretic therapy, and the degree of hypothermia was unrelated to antipyretic dosing.

Importantly, the intermittent and severe nature of hypothermia in this patient necessitated ICU‐level care, highlighting the potential for significant clinical deterioration in the absence of disease‐targeted treatment. Delay in treatment due to plans for induction chemotherapy outpatient likely prolonged her course and may have contributed to her instability.

This case adds to the small body of literature suggesting that intermittent hypothermia may serve as an early and clinically significant manifestation of HL. This case differs from previously published reports due to the severity of hypothermia and its significant impact on medical decision making. First, our patient exhibited profound intermittent hypothermia requiring ICU level care. In contrast, previously reported cases describe primarily mild hypothermia [4, 5]. In the hospital setting, recognizing lymphoma as part of the differential diagnosis in a patient with hypothermia may prompt earlier hematologic evaluation and, if the diagnosis is made, can help expedite treatment decisions in those diagnosed with hematologic malignancies. In patients with known or suspected HL who develop unexplained hypothermia, clinicians should consider urgent initiation of chemotherapy rather than deferring treatment to the outpatient setting. This approach may not only improve symptom control but also prevent complications and reduce hospital length of stay.

Overall, we feel our case has several key takeaways that can be helpful for physicians practicing hospital medicine, especially in those that take care of patients with hematologic malignancies. First, unexplained hypothermia in hospitalized patients is considered a poor prognostic indicator for many pathologies and should prompt a systemic evaluation including workup infection, endocrine, and malignant etiologies and considering medication effects and central nervous system pathology. Also, HL is a rare but significant cause of hypothermia and should be included in the differential when standard workup is unrevealing. Additionally, hypothermia in HL can occur without chemotherapy or antipyretic exposure, which suggests a possible disease‐driven mechanism such as cytokine dysregulation or autonomic dysfunction. And lastly, prompt initiation of disease‐directed therapy can lead to rapid resolution of temperature instability, and inpatient treatment should be considered in patients that are clinically unstable.

## Funding

No funding was received for this manuscript.

## Consent

No written consent has been obtained from the patient as there are no patient identifiable data included in this case report.

## Conflicts of Interest

The authors declare no conflicts of interest.

## Data Availability

The data that support the findings of this study are available from the corresponding author upon reasonable request.

## References

[1] Duong H. and Patel G. , Hypothermia, Statpearls. Treasure Island (FL), 2024, StatPearls Publishing.

[2] Zhao Y. and Zhang B. , Association Between Body Temperature and All-Cause Mortality in Patients With Sepsis: Analysis of the MIMIC-IV Database, European Journal of Medical Research. (2024) 29, no. 1, 10.1186/s40001-024-02219-2. PMC11673708 39726025

[3] Morgan M. , Schwartz L. , and Duflou J. , Hypothermia Secondary to Glioblastoma Multiforme? Autopsy Findings in Two Cases, Journal of Forensic Sciences. (2015) 60, no. 2, 511–513, 10.1111/1556-4029.12699, 2-s2.0-84924225567. 25644717

[4] Hansen J. J. , Beier Ommen H. , Gormsen L. C. , d’Amore F. A. , and Hjørnet Kamper P. M. , Classical Hodgkin Lymphoma Presenting With Severe, Recurrent Hypothermic Episodes, Case Rep Hematol.(2018) 2018, 3726593–3, 10.1155/2018/3726593. 30356349 PMC6176302

[5] Shepshelovich D. , Shpilberg O. , Lahav M. et al., Hodgkin Lymphoma and Hypothermia: Case Report and Review of the Literature, Acta Haematologica. (2014) 131, no. 4, 227–230, 10.1159/000355262, 2-s2.0-84901371790. 24335335

[6] Leon L. R. , Hypothermia in Systemic Inflammation: Role of Cytokines, Frontiers in Bioscience. (2004) 9, no. 1-3, 1877–1888, 10.2741/1381, 2-s2.0-2442708138. 14977594

[7] Bilora F. , Veronese F. , Zancan A. , Biasiolo M. , Pomerri F. , and Muzzio P. C. , Autonomic Dysfunction in Hodgkin and Non-Hodgkin Lymphoma. A Paraneoplastic Syndrome?, Hematology Reports. (2010) 2, no. 1, 10.4081/hr.2010.e8. PMC3222264 22184521

[8] Shibao C. , Muppa P. , Semler M. W. , Peltier A. C. , and Biaggioni I. , A Standing Dilemma: Autonomic Failure Preceding Hodgkin’s lymphoma, Americas Journal of Medicine. (2014) 127, no. 4, 284–287, 10.1016/j.amjmed.2013.12.002, 2-s2.0-84897885526. PMC4099002 24333616

[9] Kulkarni A. , Zlabek J. , Farnen J. , and Capla R. , Recurrent Hypoglycemia and Hypothermia in a Patient With Hodgkin’s Disease, Haematologica. (2006) 91, no. 12 Suppl. 17194656

